# Zygomycosis Associated with HIV Infection and Liver Transplantation

**DOI:** 10.4061/2011/545981

**Published:** 2011-01-23

**Authors:** Larry Nichols, Rebecca Z. Ocque, Ivonne Daly

**Affiliations:** ^1^Department of Pathology, University of Pittsburgh Medical Center, A610 Scaife Hall, 200 Lothrop Street, Pittsburgh, PA 15213-2582, USA; ^2^Department of Critical Care Medicine, University of Pittsburgh Medical Center, Pittsburgh, PA 15213-2582, USA

## Abstract

Zygomycosis is an increasing threat to patients with human immunodeficiency virus (HIV) infection. Zygomycosis (formerly called mucormycosis) is the fungal infection with *Mucor*, *Rhizopus*, or other species that share a common morphology of large empty pauciseptate hyphae with rare random-angle branching and a collapsed “twisted ribbon” appearance. Morphology allows a specific diagnosis on frozen section or smear prior to growth and identification of the fungi in culture which makes it improtant because treatment is different than that for more common mycoses such as candidiasis and aspergillosis. We present an informative and illustrative case of zygomycosis in a patient with HIV infection and liver transplantation.

## 1. Introduction

The incidence of zygomycosis (formerly known as mucormycosis) is increasing in patients with HIV infection [1]. The incidence of zygomycosis is increasing overall, especially in patients immunosuppressed for transplantation [2, 3]. Risk factors for this fungal infection include immunocompromised states, diabetes mellitus, hematologic malignancy, and neutropenia. The increasing incidence of diabetes mellitus and malignancies has been suggested as an explanation for the rise in zygomycosis in patients with HIV infection [1]. Another factor in the overall increase in zygomycosis is the use of imidazoles such as voriconazole, particularly as antifungal prophylaxis in transplant patients, because voriconazole is ineffective against Zygomycetes [2–5].

Zygomycosis is an invasive angiotropic infection with fungi of the Mucorales order, which includes *Mucor* species, *Rhizopus* species, *Rhizomucor* species, and multiple others. The most common form of infection is rhinocerebral, which is classically associated with poor glycemic control. The second most common form of infection is pulmonary and the third disseminated. Other forms of zygomycosis include cutaneous, gastrointestinal, and renal zygomycosis [6]. 

Zygomycetes have a distinctive morphology; they have large hyphae, 5 to 20 microns in width. In comparison, the hyphae of *Aspergillus* species are 3 to 5 microns in width. Zygomycetes have a very low cellular protein content, giving their hyphae an empty or hollow appearance on routine hematoxylin and eosin stain. The hyphae of Zygomycetes are pauciseptate; they have few septations. The hyphae of Zygomycetes also have few branchings, and the few they have are at random angles, including right angles and other nonacute angles. In contrast, *Aspergillus* and most other common pathogenic fungi have acute-angle branching. Because of their large size and low-protein content (relative emptiness), the hyphae of Zygomycetes are frequently collapsed in tissue sections, creating a characteristic “twisted ribbon” appearance. The distinctive morphology of Zygomycetes allows a pathologist to diagnose zygomycosis in a frozen section or smear prior to a diagnosis by culture. This is important because early diagnosis is key to better treatment outcomes and the treatment is different than that for much more common mycoses such as candidiasis and aspergillosis. The following case is informative and illustrative.

## 2. Case Report

This 50-year-old white male with a history of HIV infection diagnosed 24 years ago, having been on highly active antiretroviral therapy (HAART) for the last 9 years, had a history of intravenous drug abuse ending 21 years prior. He developed end-stage liver disease secondary to hepatitis C, presumably contracted through intravenous drug abuse. He also had a remote history of smoking and alcohol use, a remote right leg deep venous thrombosis, and chronic pancytopenia.

The patient was hospitalized with decompensated end-stage liver disease and hepatorenal syndrome. He was found to have a lupus anticoagulant. The patient developed heart failure and pulmonary edema. His CD4+ T-cell count was 108/cu mm. He improved clinically over the course of several weeks and finally was deemed a candidate for liver transplantation.

Following transplantation, he was immunosuppressed due to his renal failure with a calcineurin-sparing protocol including basiliximab (Simulect), mycophenolate, and prednisone (20 mg/day). He was also treated with daptomycin, dapsone, acyclovir, and voriconazole. His HAART was held during the first few days after transplant. Immediately after transplant, chest X-ray showed near-complete resolution of pulmonary edema and small areas of atelectasis in mid and lower left lung. That evening, the patient's temperature was 38.5 degrees, heart rate 82/minute, blood pressure 109/47 mmHg, pulmonary artery pressure 31/18 mmHg, cardiac output 13 L/min, systemic vascular resistance index (SVRI) 705, and respirations 16/minute. He was extubated.

On postoperative day 1, the patient was afebrile, but vasodilated, with blood pressure 84/52 mmHg, and norepinephrine was started. He had mild crackles on lung examination. His white blood cell (WBC) count was 600/cu mm (61% neutrophils, 4% bands, 20% monocytes, 10% lymphocytes, 5% atypical lymphocytes), hemoglobin 7.9 g/dL, platelets 28,000/cu mm, and international normalized ratio (INR) 3.3. Chest X-ray showed mild interstitial pulmonary edema and increased atelectasis at the left base. He was started on filgrastim. 

On day 2, the patient was afebrile, with respirations 26/minute, blood pressure 92/55 mmHg (on norepinephrine), cardiac output 13 L/minute, and SVRI 350. He had labored breathing. Chest X-ray showed interstitial right lung edema and a large left pleural effusion with atelectasis involving the entire left lung. The patient was placed on biphasic positive airway pressure (BiPAP), and repeat chest X-ray showed considerable expansion of the left-upper lobe. His creatinine was 3.9 mg/dL, and continuous venovenous hemodialysis was started. On day 4, the patient was intubated for mental status changes and respiratory insufficiency. He was started on hydrocortisone. On day 5, the patient required increasing doses of norepinephrine and the addition of vasopressin. Gram stain of bronchoalveolar lavage (BAL) showed rare white blood cells. Piperacillin/tazobactam (Zosyn) was added.

On day 6, the patient required mechanical ventilation with 50% oxygen. He had a “significant amount of secretions.” Computed tomography showed extensive airspace consolidation superimposed on atelectasis throughout dependent portions of the left lung associated with mucus plugging in the left-lower lobe; there was also a moderate left pleural effusion, small right pleural and pericardial effusions, and mild-moderate ascites. BAL culture from day 5 yielded >100,000/mL *Enterococcus* species. Tigecycline and caspofungin were added.

On postoperative day 7, the patient's temperature was 34 degrees, heart rate 69/minute, blood pressure 96/60 mmHg, respiratory rate 16/minute, and saturation 100% on mechanical ventilation with 40% oxygen. The patient's HAART was restarted. Vancomycin was added. His bilirubin was 3.5 mg/dL, aspartate aminotransferase (AST) 142 IU/L, lactate dehydrogenase (LDH) 672 IU/L, creatine phosphokinase (CPK) 1526 IU/L, creatinine 0.8 mg/dL, WBC count 10,300/cu mm (neutrophils 76%, bands 23%, lymphocytes 1%), hemoglobin 6.2 g/dL, platelets 33,000/cu mm, and INR 1.7. Chest X-ray showed a large left pleural effusion, airspace consolidation of the left lung and mild right perihilar interstitial pulmonary edema. Shortly before midnight, the patient suddenly developed pulseless electrical activity, and he could not be resuscitated. His glucose had been 125–180 mg/dL postoperatively. Blood culture from day 6 was later reported positive for vancomycin-resistant *Enterococcus faecium*.

**Figure 1:**
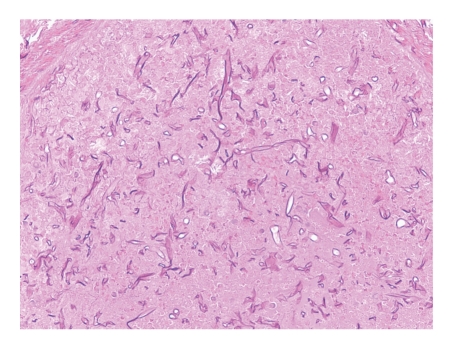
Pulmonary artery containing numerous large fungal hyphae with minimal septation and branching, some with an empty appearance (H&E, 200x).

**Figure 2:**
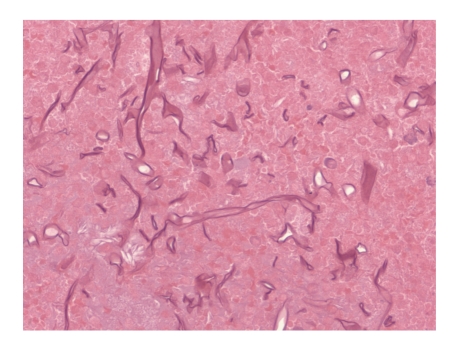
Fungal hyphae with prominent collapsed configuration resembling twisted ribbon (H&E, 400x).

**Figure 3:**
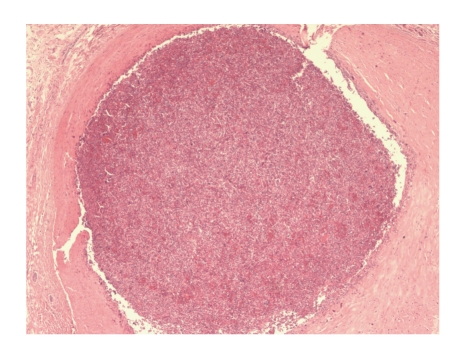
Coronary artery thrombosed with clot containing unusual basophilic strands not typical of fibrin (H&E, 40x).

**Figure 4:**
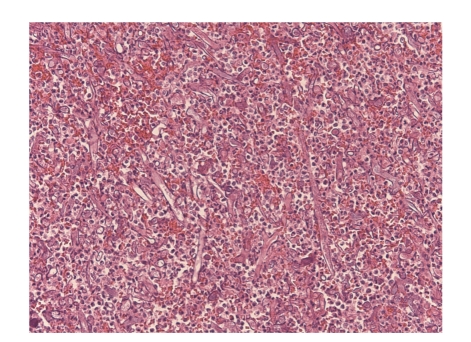
Coronary artery clot containing numerous large, relatively open, pauciseptate fungal hyphae typical of Zygomycetes (H&E, 200x).

Postmortem examination revealed massive disseminated zygomycosis involving both lungs, pericardium, coronary arteries and veins, myocardium, spleen, lymph nodes, bone marrow, stomach, and right kidney. Autopsy spleen culture was positive for *Rhizomucor* species. There was massive pulmonary consolidation, with left lung weight 1420 grams (normal up to 480 grams), and right lung weight 1130 grams (normal up to 570 grams). Autopsy revealed infected thrombosis of the main left-lower-lobe pulmonary artery with massive parenchymal zygomycosis, infarction, and hemorrhage (Figures 1 and 2). The left pleural cavity contained 1200 mL of hemorrhagic effusion. The right-upper-lobe pulmonary artery had an infected thrombus associated with extensive parenchymal zygomycosis of central right-upper lobe. There was severe coronary atherosclerosis with up to approximately 80% stenosis of the left circumflex coronary artery and infected thrombosis of left anterior descending coronary artery and multiple branches of this artery (Figures 3 and 4). Autopsy disclosed severe pericarditis with hemorrhagic pericardial effusion (105 mL). The liver showed moderate canalicular cholestasis, mild acute intrahepatic peri-cholangitis, and moderate centrolobular ischemic changes. There were 1800 mL of hemorrhagic ascites. 

Overall, the clinical history and autopsy findings in this case suggested that the cause of death was disseminated zygomycosis due to the immunosuppression required by liver transplantation for hepatitis C virus infection together with the immunocompromise of HIV infection. Coagulopathy, severe coronary atherosclerosis, and acute renal failure were regarded as contributing causes of death.

## 3. Discussion

This case illustrates the diagnostic morphologic features of zygomycosis, the difficulty of diagnosing a rare condition with nonspecific clinical manifestations, and the value of autopsy to reveal undiagnosed causes of death. With the superabundance of sophisticated and high-technology diagnostic testing available, it is widely assumed that autopsy rarely reveals any important undetected conditions, but the hypothesis that only the lack of testing leaves important conditions undiagnosed was recently tested by a study comparing the rate of finding major undiagnosed conditions at autopsy in patients who died in intensive care units, inpatient surgical units, and nursing homes [7]. The rate of finding major undiagnosed conditions was 27.8% for the medical intensive care unit patients, 32.7% for the surgery service patients, and 31.3% for the nursing home patients (*P* = .84). In addition, the investigators found no statistical difference in the complexity of workup in discrepant and nondiscrepant cases in each clinical setting. 

Autopsies of patients with HIV infection continue to disclose major undiagnosed conditions many years after the disease was first recognized. A study of 1,630 autopsies of patients with HIV infection between 1984 and 2002 found that 297 (18.2%) revealed invasive fungal infections, which were causes of death in 103 cases, and 38 of these fatal invasive fungal infections were undiagnosed antemortem [1]. In this series, zygomycosis was rare; there were no cases of zygomycosis in the 1,036 autopsies between 1984 and 1993, but there were 2 cases in the 594 autopsies between 1994 and 2002 (*P* = .42). Zygomycosis is a rare opportunistic infection in patients whose only risk factor is HIV infection.

Due to the efficacy of new combined antiretroviral therapy in the treatment of HIV infection, more of these patients are living long enough to succumb to other diseases such as hepatitis C. Transplantation has become an accepted treatment for end-stage organ disease in patients with HIV infection [8–11]. As a result, increasing numbers of patients with HIV infection are candidates for transplantation. Although one might assume that HIV-infected patients would not need immunosuppression to prevent transplant rejection, a study of 150 kidney transplant recipients with CD4+ T-cell counts above 200/cu mm found that they had a higher-than-expected rate of rejection, reaching 41% at 3 years, demonstrating the need for immunosuppression even in immunocompromised HIV-infected patients [11]. Many patients who acquired HIV infection from intravenous drug use also acquired hepatitis C via the same route, and when they develop end-stage liver disease from hepatitis C, liver transplantation becomes their hope for extended survival from their liver disease. Liver transplantation for such patients has long been performed at the University of Pittsburgh Medical Center without a disproportionate increase in opportunistic infections since 1997 [12]. Zygomycosis is an increasing, but still rare, opportunistic infection in transplant patients. In a study of 1208 invasive fungal infections among 1063 solid organ transplant recipients between March 2001 and March 2006, zygomycosis accounted for only 2% of the infections [13].

In the case we are reporting, the patient had multiple risk factors for zygomycosis: HIV infection, immunosuppression for transplantation, and neutropenia. He did not, however, have diabetes mellitus, and he had good glycemic control despite critical illness. He also did not have the most common rhinocerebral form of zygomycosis involving the paranasal sinuses, base of the skull, and brain. The clinical history and autopsy findings suggest that he developed disseminated infection starting with a pulmonary infection, but radiologic imaging of the chest and bronchoscopy with bronchoalveolar lavage failed to reveal the diagnosis. In this case, it is difficult to imagine how an antemortem diagnosis might have been made, especially early enough to alter the outcome. The first step toward making the diagnosis in other HIV patients is an awareness that zygomycosis is an increasingly likely opportunistic fungal infection in these patients. A review of zygomycosis in patients with HIV infection suggested that it is more common in those with a history of intravenous drug use and that transient neutropenia may be a critical factor in allowing this opportunistic infection to occur [14].

Successful treatment of zygomycosis generally requires intravenous amphotericin B, and surgically accessible infection with debridement improves the chances of success [4, 6, 14]. Posaconazole is a new orally administered antifungal agent with efficacy against Zygomycetes, which can play a role in effective therapy [4]. Successful therapy depends, however, on early diagnosis. Early diagnosis is possible with frozen section or smear and an astute pathologist [15].

## References

[1] Antinori S, Nebuloni M, Magni C (2009). Trends in the postmortem diagnosis of opportunistic invasive fungal infections in patients with AIDS: a retrospective study of 1,630 autopsies performed between 1984 and 2002. *American Journal of Clinical Pathology*.

[2] Cuenca-Estrella M, Bernal-Martinez L, Isla G, Gomez-Lopez A, Alcazar-Fuoli L, Buitrago MJ (2009). Incidence of zygomycosis in transplant recipients. *Clinical Microbiology and Infection*.

[3] Ambrosioni J, Bouchuiguir-Wafa K, Garbino J (2010). Emerging invasive zygomycosis in a tertiary care center: epidemiology and associated risk factors. *International Journal of Infectious Diseases*.

[4] Rogers TR (2008). Treatment of zygomycosis: current and new options. *The Journal of Antimicrobial Chemotherapy*.

[5] Severo CB, Guazzelli LS, Severo LC (2010). Chapter 7: zygomycosis. *Jornal Brasileiro de Pneumologia*.

[6] Almyroudis NG, Sutton DA, Linden P, Rinaldi MG, Fung J, Kusne S (2006). Zygomycosis in solid organ transplant recipients in a tertiary transplant center and review of the literature. *American Journal of Transplantation*.

[7] Scordi-Bello IA, Kalb TH, Lento PA (2010). Clinical setting and extent of premortem evaluation do not predict autopsy discrepancy rates. *Modern Pathology*.

[8] Stock PG, Roland ME, Carlson L (2003). Kidney and liver transplantation in human immunodeficiency virus-infected patients: a pilot safety and efficacy study. *Transplantation*.

[9] Moreno S, Fortún J, Quereda C (2005). Liver transplantation in HIV-infected recipients. *Liver Transplantation*.

[10] Di Benedetto F, Di Sandro S, De Ruvo N (2008). Human immunodeficiency virus and liver transplantation: our point of view. *Transplantation Proceedings*.

[11] Stock PG, Barin B, Murphy B (2010). Outcomes of kidney transplantation in HIV-infected recipients. *New England Journal of Medicine*.

[12] Fung J, Eghtesad B, Patel-Tom K, DeVera M, Chapman H, Ragni M (2004). Liver transplantation in patients with HIV infection. *Liver Transplantation*.

[13] Pappas PG, Alexander BD, Andes DR (2010). Invasive fungal infections among organ transplant recipients: results of the transplant-associated infection surveillance network (Transnet). *Clinical Infectious Diseases*.

[14] Nagy-Agren SE, Chu P, Smith GJW, Waskin HA, Altice FL (1995). Zygomycosis (mucormycosis) and HIV infection: report of three cases and review. *Journal of Acquired Immune Deficiency Syndromes and Human Retrovirology*.

[15] Ghadiali MT, Deckard NA, Farooq U, Astor F, Robinson P, Casiano RR (2007). Frozen-section biopsy analysis for acute invasive fungal rhinosinusitis. *Otolaryngology*.

